# A comparison between silicone‐free and silicone‐based emulsions: Technological features and in vivo evaluation

**DOI:** 10.1111/ics.12800

**Published:** 2022-08-08

**Authors:** Antonia Mancuso, Martine Tarsitano, Betty P. Udongo, Maria Chiara Cristiano, Daniele Torella, Donatella Paolino, Massimo Fresta

**Affiliations:** ^1^ Department of Experimental and Clinical Medicine Catanzaro Italy; ^2^ Department of Health Science University “Magna Græcia” of Catanzaro Campus Universitario‐Germaneto Catanzaro Italy; ^3^ Pincer Training and Research Institute Kampala Uganda

**Keywords:** cosmetic emulsions, emulsion rheology, emulsion stability, in vivo safety testing, silicones, skin feeling

## Abstract

**Objective:**

Nowadays, the use of silicones in cosmetic formulation is still controversial, given that “natural” or “biodegradable” components are preferred. Often, the exclusion and/or the discrimination of these excipients from cosmetic field are unmotivated because all things cannot be painted with the same brush. Hence, we want to bring to light and underline the advantages of including silicones in cosmetic emulsions, refuting and debunking some myths related to their use.

**Methods:**

Silicone‐free and silicone‐based emulsions were obtained within an easy homogenization process. Droplet size distribution was assessed by laser diffraction particle size analyser Mastersizer 2000™, and by optical microscopy. The long‐time stability profiles were investigated thanks to the optical analyser Turbiscan® Lab Expert. Diffusing wave spectroscopy (DWS) by Rheolaser Master™ and frequency sweep measurements by Kinexus® Pro Rotational Rheometer were carried out to assess a full rheological characterization. In vivo studies were carried out by the evaluation of Trans Epidermal Water Loss (TEWL) over time on healthy human volunteers. A skin feeling rating was collected from the same volunteers by questionnaire.

**Results:**

From size distribution analysis, a better coherence of data appeared for silicone‐based emulsion, as the size of the droplets was kept unchanged after 1 month, as well as the uniformity parameter. Morphological investigation confirmed a homogenous droplet distribution for both samples. Silicones enhanced the viscosity, compactness and strength of the cream, providing a suitable stability profile both at room temperature and when heated at 40°C. The solid‐like viscoelastic behaviour was assessed in the presence of dynamic oscillatory stresses. The monitoring of TEWL over time demonstrated non‐occlusive properties of emulsions containing silicones, the values of which were comparable to the negative control. Silicone‐based emulsions gained higher scores from the volunteers in silkiness, freshness and softness features, while lower scores were obtained in greasiness compared to silicone‐free emulsions. No cases of irritation were recorded by the candidates.

**Conclusion:**

The presence of specific silicones inside a cosmetic product improved its technological characteristics. The rheological identity and the stability feature showed the real suitability of prepared emulsion as a cosmetic product. Moreover, this study demonstrated that silicone‐based emulsions are safe for the skin and did not cause skin occlusion. Improved skin sensations are registered by potential consumers when silicones are included in the formulation.

## Résumé

**Objectif:**

De nos jours, l’utilisation de silicones dans la formulation cosmétique reste controversée, étant donné que les ingrédients «naturels» ou «biodégradables» sont privilégiés. Souvent, l’exclusion et/ou la discrimination de ces excipients du domaine cosmétique ne sont pas motivées, parce que tous les éléments ne peuvent pas être logés à la même enseigne. Par conséquent, nous souhaitons mettre en évidence et souligner les avantages de l’inclusion des silicones dans les émulsions cosmétiques, tout en réfutant et en démystifiant certains mythes liés à leur utilisation.

**Méthodes:**

Des émulsions sans silicone et des émulsions à base de silicone ont été obtenues dans le cadre d’un processus d’homogénéisation facile. La distribution des tailles de gouttelettes a été évaluée par diffraction laser avec le granulomètre Mastersizer 2000™ et par microscopie optique. Les profils de stabilité à long terme ont été étudiés grâce à l’analyseur optique Turbiscan® Lab Expert. La spectroscopie par diffusion d’ondes (Diffusing Wave Spectroscopy, DWS) par le Rheolaser Master™ et les mesures de balayage de fréquence par le rhéomètre rotatif Kinexus® Pro ont été réalisées pour évaluer une caractérisation rhéologique complète. Des études in vivo ont été menées par le biais de l’évaluation de la perte d’eau transépidermique (PETE) au fil du temps sur des volontaires humains en bonne santé. Une évaluation de la sensation cutanée a été recueillie auprès des mêmes volontaires par le biais d’un questionnaire.

**Résultats:**

L’analyse de la distribution des tailles a révélé une meilleure cohérence des données pour l’émulsion à base de silicone, car la taille des gouttelettes a été maintenue inchangée après 1 mois, ainsi que le paramètre d’uniformité. L’investigation morphologique a confirmé une distribution homogène des gouttelettes pour les deux échantillons. Les silicones ont amélioré la viscosité, la densité et la résistance de la crème, offrant ainsi un profil de stabilité approprié aussi bien à température ambiante qu’après chauffage à 40°C. Le comportement viscoélastique analogue à celui d’un solide a été évalué en présence de contraintes oscillatoires dynamiques. Le suivi de la perte d’eau transépidermique (PETE) au fil du temps a établi des propriétés non occlusives des émulsions contenant des silicones, dont les valeurs étaient comparables à celles du contrôle négatif. Les émulsions à base de silicone ont obtenu des scores plus élevés chez les volontaires en termes de caractéristiques de douceur, de fraîcheur et de souplesse, tandis que des scores plus faibles ont été obtenus en termes d’onctuosité par rapport aux émulsions sans silicone. Aucun cas d’irritation n’a été enregistré chez les candidats.

**Conclusion:**

La présence de silicones spécifiques dans un produit cosmétique a amélioré ses caractéristiques technologiques. L’identité rhéologique et la caractéristique de stabilité ont montré la pertinence réelle d’une émulsion préparée en tant que produit cosmétique. De plus, cette étude a démontré que les émulsions à base de silicone sont sans danger pour la peau et n’ont provoqué aucune occlusion cutanée. Les consommateurs potentiels enregistrent une amélioration des sensations cutanées lorsque des silicones sont inclus dans la formulation.

## INTRODUCTION

Silicone (e.g. poly[dimethyl siloxane] and derivatives) polymers are widely commercialized polymers used in several fields such as cosmetics [1, 2, 3]. They are considered as a class of hybrid organic/inorganic compounds able to improve physical characteristics of some commercial products. Silicone‐based cosmetics show several beneficial effects as a function of type and chemical structures of silicones used for preparation. Generally, the addition of silicone in cosmetic products, above all cosmetic emulsions, is motivated by the resulting pleasant velvety soft feeling. Dimethicone, that is the main exponent of this ingredient class, is able to exercise emollient, antifoaming and protective effects [4, 5]. Dimethicone and its derivates are approved by the Food and Drug Administration for their use and they are considered as safe ingredients by the European Commission for use in cosmetics [6]. Nevertheless, in the last decades a “witch hunt” was carried out against cosmetic products containing silicones, only because of their not‐totally natural origin [7, 8]. Many cosmetic companies, in order to attract more consumer attention to their products, have begun to align themselves with the concept of “cleaner environment”, advertising their products as “chemical‐free”, “green”, “natural” and so on [9]. Of course, silicones are poorly biodegradable, and their accumulation could be considered as a long‐term risk for the environment, but in many cases this concept has been taken to extremes, leading to the belief that silicones are totally toxic not only for the environment but also for human health [10, 11]. Hence, the labels of many cosmetic products have started to present the words “silicone‐free” leading the consumer to think that the mentioned product is safer than those containing silicones. Actually, several scientific works demonstrated that silicones are suitable even for sensitive skin as they are non‐irritating, hypoallergenic, non‐comedogenic, non‐occlusive and odourless. Silicones can be found in auxiliary cosmetic products for the treatment of eczema, acne or in products for baby care as in creams useful for soothing diaper rash [12, 13]. Moreover, it has been demonstrated that the allergic reactions consequent of silicone use are very rare [14, 15].

From a technological point of view, some silicones, such as dimethicone and its derivates, are very useful in emulsion preparation, despite their high cost [16, 17, 18]. They can guarantee a good stability and a suitable rheological profile, as well as to improve the skin feeling of resulting emulsion.

Through this research work we want to help dispel some of the criticisms attributed to silicones. For this purpose, we prepared two simple O/W emulsions with and without silicones and, after a deeper technological and formulative characterization, they were tested by applying on human healthy volunteers' skin, evaluating the occlusive effect of resulting emulsions and collecting the impressions of potential consumers on the primary and secondary skin feeling. The feedback of human volunteers, considered as potential consumers, is very important for any cosmetic company, as well as the evaluation of occlusive skin condition that could occur after silicone‐based emulsion application. Occlusion condition could be required in some situations, as for impermeabilizing the skin, for reducing the physiological dehydration and for no‐transfer effects [19, 20, 21]. However, the occlusion of skin could induce alteration in skin structures, comedones formation and alteration of microbial flora [22, 23]. One of the main parameters that provide information on the occlusive effect of a specific component is the Trans Epidermal Water Loss (TEWL) measurement, which permits to monitor the flux of physiological water from skin to external environment. TEWL is a fundamental parameter also to evaluate the integrity of the stratum corneum, since an increased TEWL value can correspond to a reversible or irreversible damage of skin, causing a freer release of water [24]. Starting from these assumptions, any variation of TEWL values was also considered during the study, following the administration of silicone‐free or silicone‐based emulsions to investigate their effect in terms of occlusive effect.

## MATERIALS AND METHODS

### Materials

Arlamol™ PS15E (Polypropylene Glycol 15 stearyl ether) and Arlacel™ 985 (Steareth‐2 [and] PEG‐8 Distearate) were purchased from Croda International Plc (Snaith, UK) and Croda Iberica SA Mevisa (Barcelona, Spain) respectively. Glycerol (1,2,3‐Propanetriol) for molecular biology, ≥99.0% was purchased by Sigma Aldrich (St. Louis, MO, USA). KSG‐210 (Dimethicone [and] Dimethicone/PEG‐10/15 Crosspolymer) and KF‐96 L‐2cs (Volatile Dimethicone) by Shin‐Etsu Chemical Co., Ltd were purchased from Prodotti Gianni SRL (Milan, Italy). Kemipur 100 (Imidazolidinyl Urea, N,N″‐Methylenebis[N′‐[3‐[hydroxymethyl]‐2,5‐dioxo‐4‐imidazolidinyl]‐urea) was obtained from A.C.E.F. SpA (Fiorenzuola D'Arda, Piacenza, Italy). Vaseline oil was purchased from POLICHIMICA SRL (Bologna, Italy). Double‐distilled milli‐Q water was used in all the experimental procedures and all other chemicals, reagents and solvents were received from commercial sources and were of analytical grade.

### Preparation of oil‐in‐water (O/W) emulsions

Oil‐in‐water emulsions were prepared by homogenizing 85 wt % aqueous phase with 15 wt % oil phase. The aqueous phase was made up by Glycerol and Arlacel™ 985, while Arlamol™ PS15E was the main component of the oil phase (Table 1). Both solutions (oil and aqueous phases) were heated at 65 ± 1°C, under constant stirring at 250 rpm. When complete solubilization of components in each phase was achieved and the temperature was stable, the oil phase was added to the aqueous phase under homogenization at 1500 rpm by the high shear mixer Silverson® L4TR. The mixing was carried out until a homogeneous emulsion was obtained. Finally, the emulsions were left cooling to room temperature, and a preservative Kemipur 100 was added. The same protocol was performed to prepare O/W emulsions in the presence of silicones. In this case KSG‐210 and KF‐96 L‐2cs were added to the oil phase together with Arlamol™. The exact composition of silicone‐free and silicone emulsions is reported in Table 1.

**TABLE 1:** Composition in weight percentage (wt %) of two types of O/W emulsions

		Silicone‐free emulsion	Silicone‐based emulsion
	Component	wt %	
	Arlacel™ 985	8	8
Aqueous phase	Glycerol	4	4
	Water	72.8	72.8
	Arlamol™ PS15E	15	7
Oil phase	KSG‐210	‐	3
	KF‐96 L‐2cs	‐	5
Preservative	Kemipur 100	0.2	0.2

All steps of characterization were carried out after 24 h of emulsification, to ensure a suitable equilibration of the emulsions [25, 26].

### Size distribution

Droplet size of emulsions was defined by a laser diffraction particle size analyser Mastersizer 2000™ (Malvern Instruments, Worcestershire, UK), equipped with Hydro 2000MU as dispersion unit for wet dispersions. Determinations were performed in triplicate. The pump speed was set at 1500 rpm and milli‐Q water was used as dispersant [27]. Before starting the analysis, the dispersant was left to equilibrate at room temperature for at least 30 min, then it was sonicated for 2 min to avoid the presence of air bubbles [28]. Each emulsion was added dropwise until the laser obscuration reading reached a value between and no further than 10%–20% of obscuration, as required by the instrument [29]. The analysis was repeated after 1 month from the preparation's date to have a check on emulsions' stability over time. The parameters recorded by MasterSizer 2000 Software v5.60 (Malvern Instruments Ltd., Worcestershire, UK) were the average particle diameter, expressed as volume‐surface mean diameter (*D*_*3,2*_) and volume‐weighted diameter (*D*_*4,3*_), uniformity and span values, meaningful for droplet polydispersity. The span value is calculated following the equation:

$$\text{Span}=\frac{D_{0.9}-D_{0.1}}{D_{0.5}}$$

where D_0.9_, D_0.5_ and D_0.1_ are the diameters of droplets below which 90%, 50% and 10% of the sample lies, respectively.

### Optical microscopy

The structures of emulsions were observed with Morphologi G3‐S microscope equipped with the optical system Nikon® CFI 60 Brightfield/Darkfield. For each sample analysed a drop of emulsion (as no‐diluted sample or diluted sample) was placed on a microscope slide and then covered with a cover slip (20 × 20 mm, Syntesys, Padova, Italy) to obtain a thin layer. Photographs of dispersed droplets' morphology were captured using 50× and 20× magnifications and finally exported as TIFF images by means of Morphologi software (Malvern Panalytical, Worcestershire, UK).

### Diffusing wave spectroscopy (DWS)

The Rheolaser Master™ (Formulaction, L'Union, France) was used to assess a microrheology examination of free‐silicone and silicone emulsions by means of diffusing wave spectroscopy (DWS). To evaluate variations in microrheology with temperature, a sample of each emulsion (20 mL) was put into suitable thermostated vials, and a full characterization analysis was carried out for 2 h at 25 ± 1°C and 40 ± 1°C, to simulate an extreme storage condition. The results were interpreted in terms of mean square displacement (MSD) of particles, due to their Brownian motion, and macroscopic viscosity index (MVI). Each specific parameter processed by RheoSoft Master 1.4.0.0. Software (Formulaction, L'Union, France) provided a contribution in describing viscoelastic properties of samples [30].

### Dynamic rheology studies

Rheological measurements of samples were obtained with Kinexus® Pro Rotational Rheometer (Malvern Panalytical Ltd., Worcestershire, UK) and the results were elaborated by means of rSpace Software v.1.60.1731. After emulsification, the samples were stored at room temperature for 24 h in sealed glass vials. After loading on the measurement plate, each sample was maintained at rest for 5 min to reduce any effect of loading on sample structure. Stress sweep tests (1 Hz at 25°C) were carried out with the only purpose of determining the linear viscoelastic region (LVER) in which to operate later [31]. Oscillatory frequency sweep measurements were then performed inside LVER and by setting a frequency range of 0.1–10 Hz and a constant shear stress of 1.0 Pa [32]. The analysis was carried out at 25 ± 1°C [33] and 40 ± 1°C onto two aliquots of the same sample, using a stainless‐steel cone‐plate geometry (diameter 40 mm; angle 2°, gap size of 1 mm) and mechanical parameters such as shear moduli (elastic and viscous components, G′ and G″) and shear viscosity (complex component, ɳ*) were recorded. All measurements were performed in triplicate.

### Stability studies

The stability of obtained emulsions was determined firstly by macroscopic examination, evaluating any phase separation process, and then more accurately by using a Turbiscan Lab® Expert (Formulaction, L'Union, France), equipped with a Turbiscan Lab Cooler. After 24 h from emulsification, a stability study over time was performed on each obtained emulsion (in the presence and in the absence of silicones) at 25 ± 1°C and 40 ± 1°C for 1 h [34]. The sample was placed into a suitable cylindrical glass tube, then it was left equilibrating at a chosen temperature for 10 min. During the analysis, the integrated TurbiSoft Lab software v.2.3.1.125 (Formulaction, L'Union, France) recorded variations in delta‐backscattering (ΔBS) and delta‐transmission (ΔT) profiles, which can highlight phenomena of emulsions' instability such as creaming, flocculation or phase separation [35]. The variation in the ΔBS and ΔT signal was estimated as the difference among the recorded backscattering/transmission signals at each scanning time and the same values documented by the instrument at time 0, as reported by the equation

$$∆Y=Y_{t_{x}}-Y_{t_{0}}$$

where Y could represent backscattering (BS) or transmission (T), t_x_ represents each time at which the instrument operated a single and full scan on sample, and t_0_ represents the scan made at the beginning of the analysis. The values of ΔBS and ΔT were plotted as mean values ± standard deviation versus the height of the sample present in the glass vial.

### In vivo trans epidermal water loss (TEWL) evaluation

C + K Multi Probe Adapter equipped with probe Tewameter® TM300 (Courage & Khazaka, Cologne, Germany) was employed to carry out an in vivo evaluation of TEWL (Trans Epidermal Water Loss) [36]. The water evaporation gradient through the skin was evaluated before by topically applying pure silicones and then by spreading on the skin sites both prepared emulsions, in the presence of and without silicones.

For these studies human healthy volunteers (*n* = 12, mean age, 25 ± 5 years; female sex) were enrolled. They were adequately informed about the nature and characteristics of the study, and then they signed informed agreements. The in vivo studies were carried out in accordance with the Declaration of Helsinki. The protocol was approved by the Research Ethics Committee of the University of Catanzaro “Magna Græcia” (Approval numbers: 391/2019 and 392/2019). Before starting the analysis, the volunteers were accommodated and acclimatized under controlled experimental conditions of room temperature (25 ± 1°C) and relative humidity (50%). To carry out experiments in a non‐occlusive condition, commercial patches for non‐occlusive tests (Farmacosmo S.r.l., Napoli, Italy) were used.

Four sites of 1 cm^2^ were identified on the left forearm of healthy human volunteers in the absence of skin discoloration and comedones. The first site was treated with 200 μL of NaCl 0.9% (w/v) aqueous solution, used as the negative control of the occlusion. The second site was treated with 200 μL of Vaseline oil, used as the positive control of the occlusion, while on the remaining sites equal amounts of KF‐96 L‐2cs and KSG‐210 were applied as free ingredients.

Instead, three sites of 1 cm^2^ each on the right forearm of each volunteer were isolated. The first site was treated with 200 μL of NaCl 0.9% (w/v), while the second and the third sites were treated with 200 mg of emulsions with and without silicones respectively. TEWL values were determined after 0, 1, 2, 4, 6, 8 h from application [37].

### Skin feeling

The same volunteers enrolled for previous in vivo studies did agree to rate the emulsions, describing the primary and secondary skin feelings [38], respectively, during and after spreading 200 mg of samples (in the absence and in the presence of silicones) on the back of their hands. The procedure was performed in single blind, and therefore only the operator was aware of the differences between the two applied products. The volunteers' feedback was collected through an anonymous questionnaire with rating values from −5 to +5 about softness, heaviness, spreadability and freshness (primary skin feeling) and moisturizing sense, silkiness, irritation or greasiness (secondary skin feeling) [39]. The values, from lowest to highest, indicated, respectively, a rating from “very poor/absent” to “very good/excellent” in association with product skin feeling.

### Statistical analysis

The statistical analysis was carried out with a one‐way ANOVA test. Bonferroni t‐test was used to check the obtained results and the significance levels were fixed at *p < 0.05 and **p < 0.001.

## RESULTS AND DISCUSSION

### Physicochemical characterization of emulsions

Silicone‐based and silicone‐free emulsions were obtained through an easy‐to‐manufacture process, taking advantage of high shear homogenization. Defining the size distribution and morphology of the oil droplets dispersed in the aqueous medium is a crucial aspect in the characterization of emulsifying systems. Indeed, the size and the concentration of the oil in water dispersed droplets can strongly influence the stability of the emulsion obtained, as well as its rheology [40]. The evaluation of these parameters allows us not only to characterize an emulsion and to define it in its proper class but also to predict possible destabilization phenomena such as creaming or phase separation. After preparation, the formulations were sealed and left to rest for 24 h, to ensure a suitable systems equilibration before proceeding to the physico‐chemical characterization.

**FIGURE 1:**
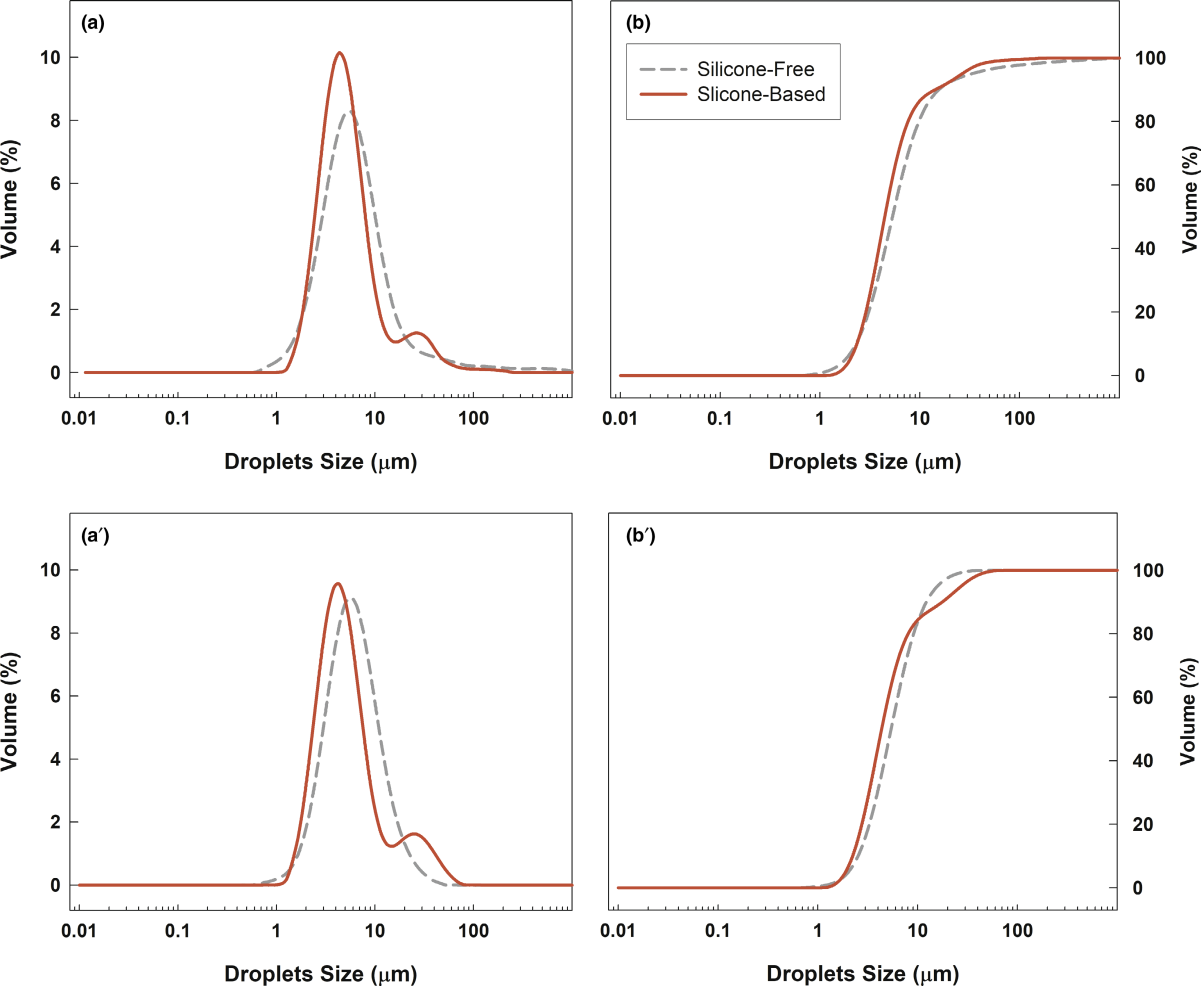
Size distribution curves (panel a) and cumulative under size curves (panel b) of silicone‐free and silicone‐based emulsions. Panels a′ and b′ are related to the same analysis repeated after 1 month from the preparation's date. The results are representative of three independent experiments [Colour figure can be viewed at wileyonlinelibrary.com]

The droplet size analysis, assessed by laser diffraction method, revealed different size distribution curves for emulsions prepared with or without silicones. As shown in Figure 1a, in the first analysis, carried out after 24 h, both emulsions had a good size distribution, without interfering peaks, but the red bimodal curve of volume fraction (%) versus droplets size (μm) was slightly shifted to the left, highlighting a smaller volume median diameter (D_0.5_) of silicone‐based emulsion than the counterpart without silicones (grey curve).

The D_0.5_ value is considered a reference standard for the dimensional analysis of particles because it represents the median value below and above which the entire particle population belongs [41]. The recorded D_0.5_ values were 5.34 ± 0.14 μm and 4.46 ± 0.08 μm for silicone‐free and silicone‐based emulsions, respectively, and this reduction was statistically significant (**p˂0.001). The cumulative undersize curves of formulations exhibited noticeable slopes as well as an almost complete overlap of plots, confirming a homogeneous distribution of the oil droplets dispersed in water both for silicone‐free and silicone‐based emulsions (Figure 1b).

Other parameters derived from the dimensional analysis must also be taken into consideration such as the volume‐surface mean diameter (*D*_*3,2*_) and the volume‐weighted mean diameter (*D*_*4,3*_). Each of these diameters has a different meaning in size distribution understanding. *D*_*3,2*_ is more sensitive to the presence of small particles, while *D*_*4,3*_ is the most sensitive value to the presence of large particles and consequently it provides a better estimation of droplets' aggregation in emulsion [25]. *D*_*3,2*_ values at 24 hours from sample preparation were relatively small for both types of emulsions, but the data related to silicone‐based emulsion resulted significantly lower (*p ˂ 0.05) than silicone‐free ones. Also, *D*_*4,3*_ values showed the same trend, since silicone‐based emulsion presented a lower but non‐statistically significant *D*_*4,3*_ value with respect to the silicone‐free one, therefore this could represent an advantage for formulation's stability (Table 2). Using MasterSizer 2000™ for laser diffraction analysis, the value of uniformity can clarify how symmetrical the size distribution around the median point is, while span's value gives information about the width of distribution, comparable to the conventional polydispersity index [42]. The silicone‐based emulsion showed a lower uniformity value than its counterpart, giving a confirmation of a better symmetry of size distribution. Furthermore, considering that the smaller the span value the narrower size distribution, the presence of silicones did not alter this feature. After 1 month the emulsions were analysed again in terms of droplet size distribution, to assess their stability over time [43].

**TABLE 2:** Parameters recorded by MasterSizer 2000™ affecting the size distribution of samples

	D_3,2_ (μm)	D_4,3_ (μm)	D_0.1_ (μm)	D_0.5_ (μm)	D_0.9_ (μm)	Span	Uniformity
24 h							
Silicone‐free	4.35 ± 0.05	15.41 ± 7.35	2.30 ± 0.09	5.34 ± 0.14	16.19 ± 3.63	2.59 ± 0.63	2.23 ± 1.35
Silicone‐based	4.12 ± 0.05	7.69 ± 1.38	2.34 ± 0.08	4.46 ± 0.08	14.51 ± 4.08	2.72 ± 0.88	1.03 ± 0.30
1 month							
Silicone‐free	4.53 ± 0.02	6.69 ± 0.01	2.53 ± 0.01	5.42 ± 0.03	12.23 ± 0.04	1.79 ± 0.02	0.58 ± 0.01
Silicone‐based	4.02 ± 0.03	7.34 ± 0.19	2.23 ± 0.01	4.36 ± 0.03	17.60 ± 0.94	3.52 ± 0.19	1.00 ± 0.03

*Note*: The results are reported as mean values ± standard deviation of three independent experiments.

From Table 2 it is easy to understand that the mean sizes of emulsions were retained after 30 days of storage in room temperature condition without substantial changes. D_3,2_ values of both emulsions showed significant reductions with respect to the same samples analysed 1 month earlier (*p ˂ 0.05) as well as the considerable decrease in D_0.5_ that silicone‐based sample showed against the silicone‐free emulsion was maintained with the same significance value (**p ˂ 0.001).

Comparing the results 1 month after the preparation of the emulsions, the variations of two parameters are striking. Indeed, instead of the silicone‐based emulsions which maintained almost unchanged D_4,3_ values, a remarkable difference in the same parameter of silicone‐free sample occurred.

Also, in the estimation of uniformity values, the silicone‐free sample showed a reduction after 1 month compared with the one recorded after 24 h. On the contrary, the silicone‐based sample upheld a uniformity value, which was not altered by time.

At first glance, this evidence would seem to favour the silicone‐free emulsion, because at 1 month it achieved a good stability value, even lower than the silicone counterpart. However, the event to underline is that the silicone‐free emulsion clearly requires much more time to achieve this stability in terms of the distribution's homogeneity. On the contrary, the silicone one, which was already characterized by a suitable size distribution after 24 h (1.03 ± 0.30), kept it unchanged when analysed after 1 month (1.00 ± 0.03).

Based on the emerging features, the silicone‐based emulsion could be considered as more convenient formulations, thanks to the fact that the parameters evaluated did not undergo any changes over time.

Even if the size distribution results appeared promising, employing several analytical techniques is always the best way to obtain a more realistic characterization of a specific feature. In this aim, the microstructures of emulsions were observed by an optical microscope, by using 20× and 50× magnifications.

**FIGURE 2:**
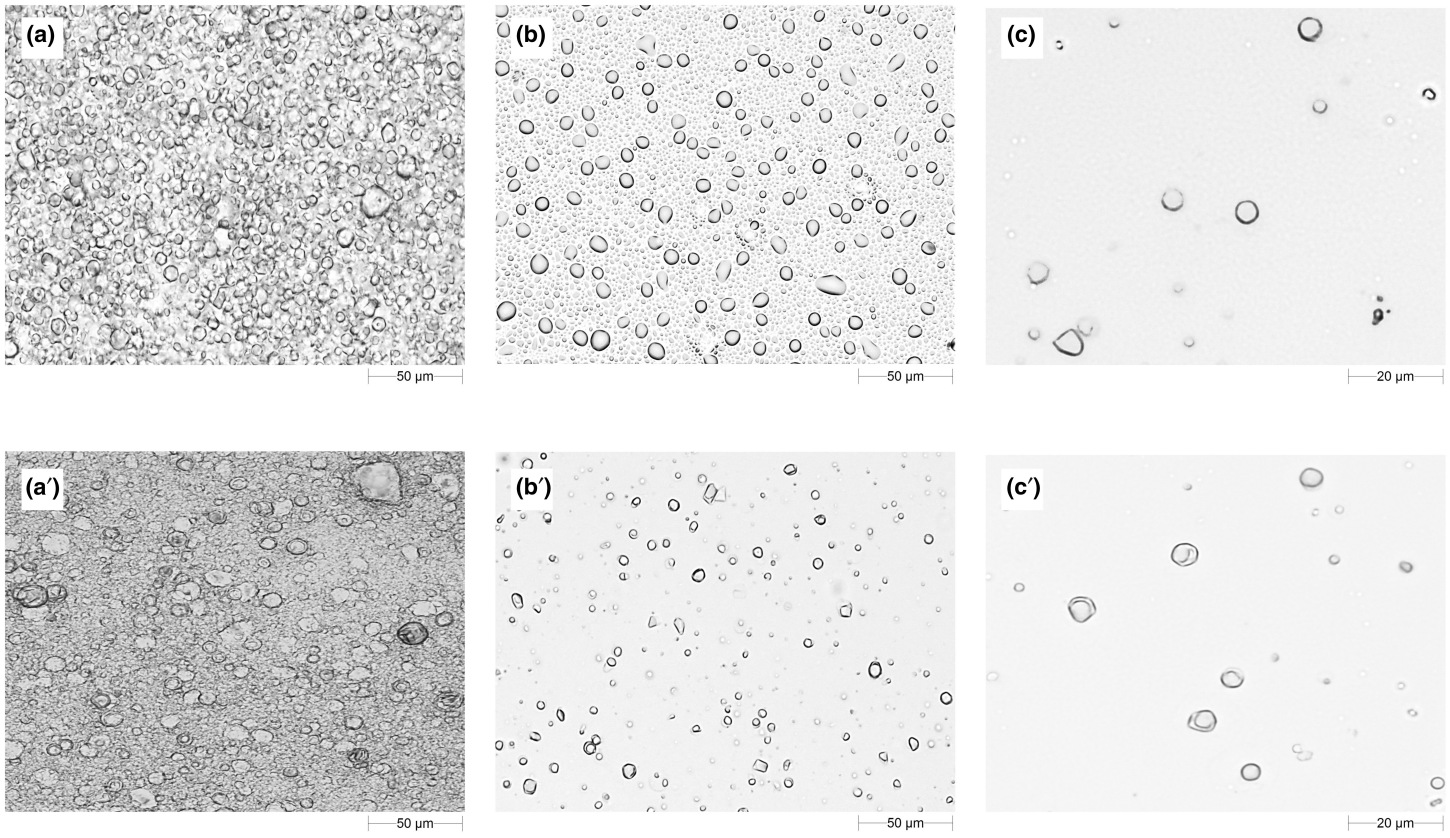
Microstructures of silicone‐free emulsions (panels on the top row) and silicone‐based emulsions (panels on the bottom row) obtained by optical microscopy. Panels a and a′ refer to undiluted samples (magnification 20×), panels b and b′ (magnification 20×) and panels c and c′ (magnification 50×) refer to the diluted sample

Initially, when the samples were analysed in their original form (Figure 2, panels A and A′) the droplets appeared overlayed, and it was difficult to see them distinctly. For this reason, one drop (approximately 80 mg) of each sample was diluted in 15 mL of MilliQ water and gently shaken for 1 min. Then one drop of diluted emulsion was placed between two glass coverslips and observed using an optical microscope.

The obtained micrographs confirmed the results highlighted by size distribution analysis, especially in terms of homogeneity of distribution of the dispersed droplets, which was not influenced by the presence of silicones in the formulation. This effect was better visible under 50× magnification (Figure 2, panels C and C′).

The study of emulsions' stability includes a huge variety of investigations and techniques, among which the dimensional distribution of the dispersed droplets represents only a small piece. In fact, especially with regard to O/W emulsions, widely used as cosmetic products, several methods have been proposed to assess stability, including light scattering and droplet size analysis [44, 45].

In the cosmetic field, the stability of the emulsions is a pivotal point not only in the development stage or to ensure the quality of the final product but also to make them highly marketable [46].

It is well known that the worst enemies of emulsions could be creaming, flocculation and/or phase separation. We evaluate organoleptic characteristics and homogeneity of formulations over time to identify visible instability like creaming, flocculation or coalescence and until 30 days from the preparation date neither sediments nor separations were visible.

Even if the macroscopically investigation could exclude some of the aforementioned phenomena, we report a stability study on emulsions carried out with Turbiscan Lab® Expert, an optical scanning instrument which is able to detect any destabilizing phenomena on the systems thanks to its continuous detection from top to bottom along the entire sample under examination [47]. We carried out the investigation first at room temperature (25 ± 1°C) and then at 40 ± 1°C, to mimic a storage in extreme heating conditions.

**FIGURE 3:**
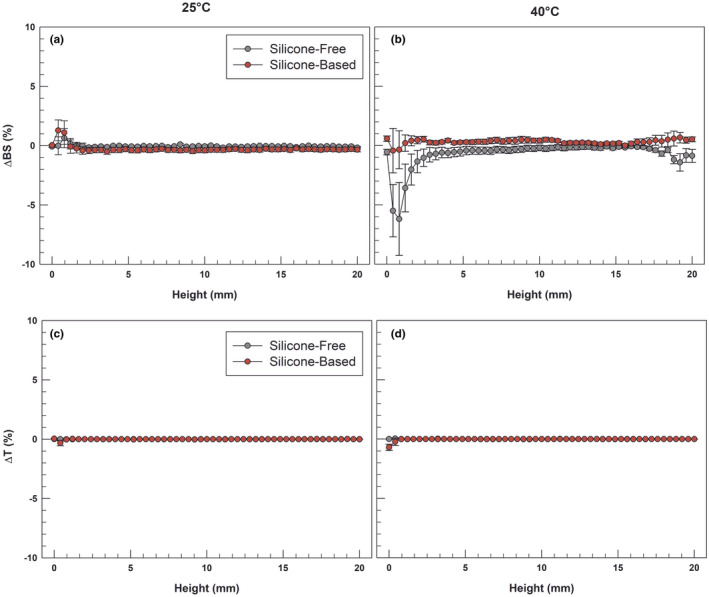
Delta‐backscattering (ΔBS, panels a and b) and delta‐transmission (ΔT, panels c and d) profiles of emulsions at 25 ± 1°C and 40 ± 1°C. The results are reported as mean values ± standard deviation of three independent experiments [Colour figure can be viewed at wileyonlinelibrary.com]

The multiple light scattering analysis provided delta‐backscattering and delta‐transmission profiles of both the emulsions. As shown in Figure 3 at 25°C there was no difference between two samples both in delta‐backscattering and in delta‐transmission profiles since the curves appeared overlayed.

Therefore, the presence of additional silicone components does not involve alterations in stability profiles, and this allowed us to consider the two formulations entirely comparable in terms of stability at a temperature of 25°C.

When subjected to the same analysis but increasing the temperature to 40°C, the backscattering profile of the silicone‐free emulsion slightly differed from the previous in the final range about 18–20 mm of height, while the formulation containing the silicones proved to be unperturbed by the heat increase (panel B).

This effect could be a synonym of greater compactness and strength of the silicone cream, which can better keep its structure intact than the free one without undergoing destabilizing phenomena.

Overall, both the formulations showed good ΔBS and ΔT values, which never exceeded ±2%, with the exception of the first millimetres of silicone‐free sample in panel B, whose destabilization is not related to the system instability but could be attributable to the thickness of the bottom of the cylindrical vial [48].

Another stage in defining the stability of the emulsions belongs to the analysis of the rheological features, which can not only predict destabilization phenomena but can also give information about changes in the emulsion structure as a function of external conditions, such as temperature [49].

The comparison between two emulsions went ahead with a deeper microrheological investigation by using Rheolaser Master™, which represents an evolution of dynamic light scattering technique and allows the analysis of formulations at rest, even without mechanically stressing, but modifying some parameters such us temperature [50]. Diffusing Wave Spectroscopy (DWS) is the theory on which the operation of the instrument is based, and it represents a compelling technique for analysing very turbid formulations, such as our emulsions, in a non‐destructive way also allowing a total recovery of the sample itself [51].

At the light of these considerations, we fully characterized the microrheological behaviour of emulsions, both at 25°C and rising temperature to 40°C, leveraging the instrument's capability to detect and monitor changes in sample stability in real time. The first parameter investigated was the Mean Square Displacement (MSD), which permits quantifying the Brownian movements of emulsion droplets, considering that the movement freedom is correlated with the internal structure of samples [52]. The MDS curves enabled the characterization of the viscoelastic properties of the sample. In detail, when a sample is characterized by a low passive viscosity, the relative MSD curves grow linearly with decorrelation time as the internal particles are completely free to move. On the contrary, when the sample is characterized by a more viscoelastic behaviour, the MSD curves assume a non‐linear shape as the particles are not free to move due to the presence of a denser inner network. The microrheological analysis allows to compare the inner structure of two or more samples, which seem apparently analogous.

**FIGURE 4:**
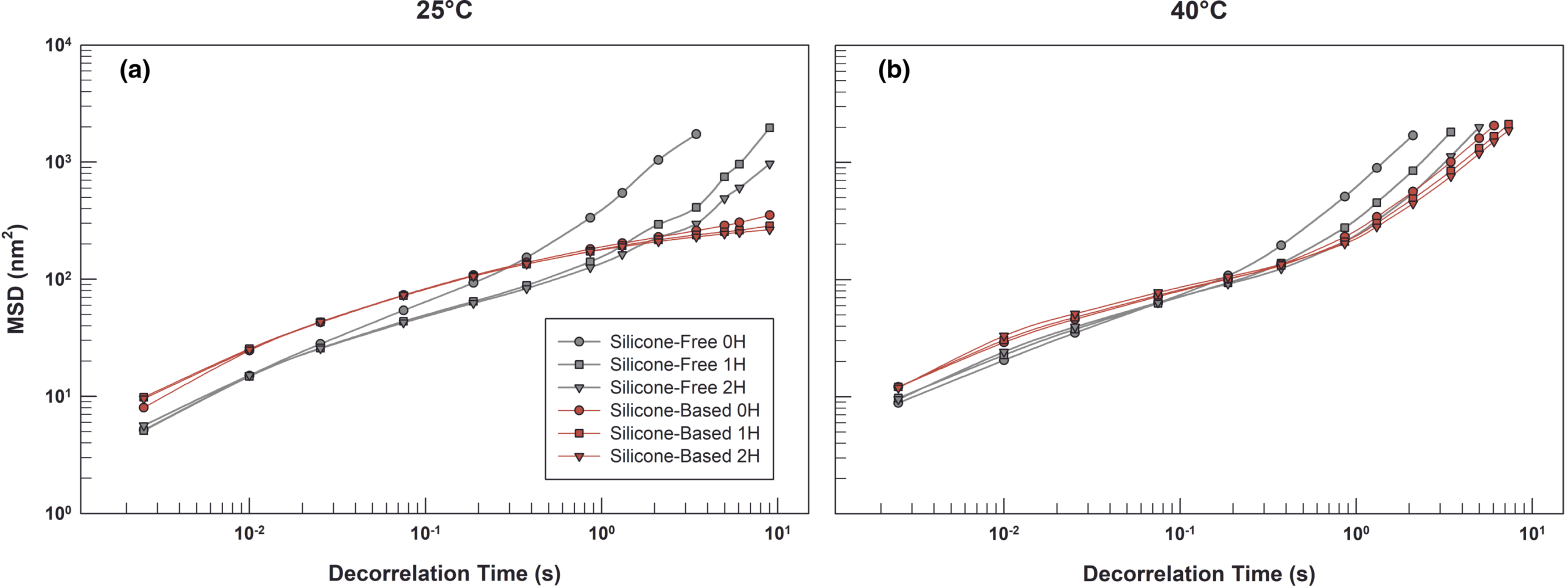
Mean square displacement (MSD) of silicone‐free and silicone‐based emulsions at 25°C ± 1°C (panel A) and 40°C ± 1°C (panel B) as a function of decorrelation time. The results are representative of three independent experiments. [Colour figure can be viewed at wileyonlinelibrary.com]

As shown in Figure 4, when the analysis was carried out at 25°C, the two emulsions revealed their different microrheological behaviour.

In fact, while the droplets of silicone‐free sample were more subjected to Brownian motions, the ones belonging to the silicone‐based emulsion appeared unperturbed by the DWS analysis, as perceived by the almost complete superimposition of the red curves, even at different times.

Therefore, the absence of changes in slopes of the red curves and the rapid achievement of a plateau are signals that the silicone‐based emulsion droplets are limited in movement. Moreover, the limitation of movements is due to a tighter internal structuring of silicone‐based emulsion. This could be ascribable to the presence of KSG‐210, an emulsifying silicone elastomer that belongs to the class of dimethicone cross‐polymers. In fact, because of its chemical structure it is officially recognized and used as an increasing‐viscosity agent, and therefore in this formulation it was able to induce the formation of a tighter three‐dimensional network that maintains blocked the emulsion droplets [15], limiting their movements within the sample. When the system was heated to 40°C, the difference in slopes among grey plots was maintained, while, even if the red plot showed an increase in slopes, the silicone‐based sample still showed an overlapping of MSD curves at different times of analysis.

This result was not unexpected, as it is quite usual that by increasing the temperature a decrease in viscosity occurs and consequently an improvement in the freedom of movement of the dispersed oil particles can be recorded.

The parameter of Macroscopic Viscosity Index (MVI), recorded by microrheology, is related to the inverse MSD slope in linear scale and it is well known its link in meaning with macroscopic or complex viscosity (η*), determined in Pa·s [47]. Even if the investigated parameters are strongly dependent on the type of analysis performed and they differ primarily in the dimensionality of the values, when combined, their result's meaning can provide a complete interpretation of samples' behaviour.

In this study, we also carried out a dynamic rheological characterization, by means of the Kinexus® Rotational Rheometer to understand if, following dynamic stresses, the viscoelastic behaviour of the samples could deviate from the results obtained by microrheology investigation under static conditions.

We evaluated mechanical properties of samples, such as the elastic and the viscous moduli (G′ and G″ respectively) and complex viscosity (η*), within a low frequency range and by means of a constant shear stress, fixed at 1 Pa.

**FIGURE 5:**
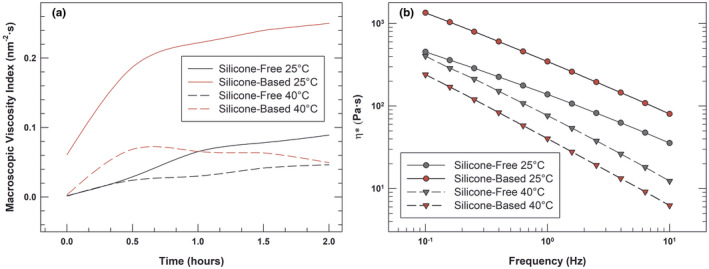
Macroscopic Viscosity Index (MVI, panel a) profiles versus time compared with complex viscosity (η*, panel b) profiles versus frequency at 25°C ± 1°C and 40°C ± 1°C. The results are representative of three independent experiments [Colour figure can be viewed at wileyonlinelibrary.com]

In Figure 5 we reported a comparison between the viscosity profiles of the emulsions obtained by a static rheology investigation (panel A) and by the frequency sweep measurement with dynamic rheometer (panel B).

After all, the observed behaviour under static conditions, where only a thermal stress is applied, could be quite different when an oscillatory mode stress is applied. In both static and dynamic conditions, at 25°C the silicone‐based viscosity curves predominated over the silicon‐free ones, and the viscosity of both samples decreased with heating.

Although the viscosity dependence of silicone‐based emulsion from temperature clearly emerged through the microrheological analysis by means of MSD evaluation and MVI (panel A), it was accentuated when the sample was simultaneously subjected to thermal stress and oscillatory stress with the dynamic investigation (panel B).

A further study of the viscoelastic profiles of the emulsions at 25 and 40 °C was assessed.

**FIGURE 6:**
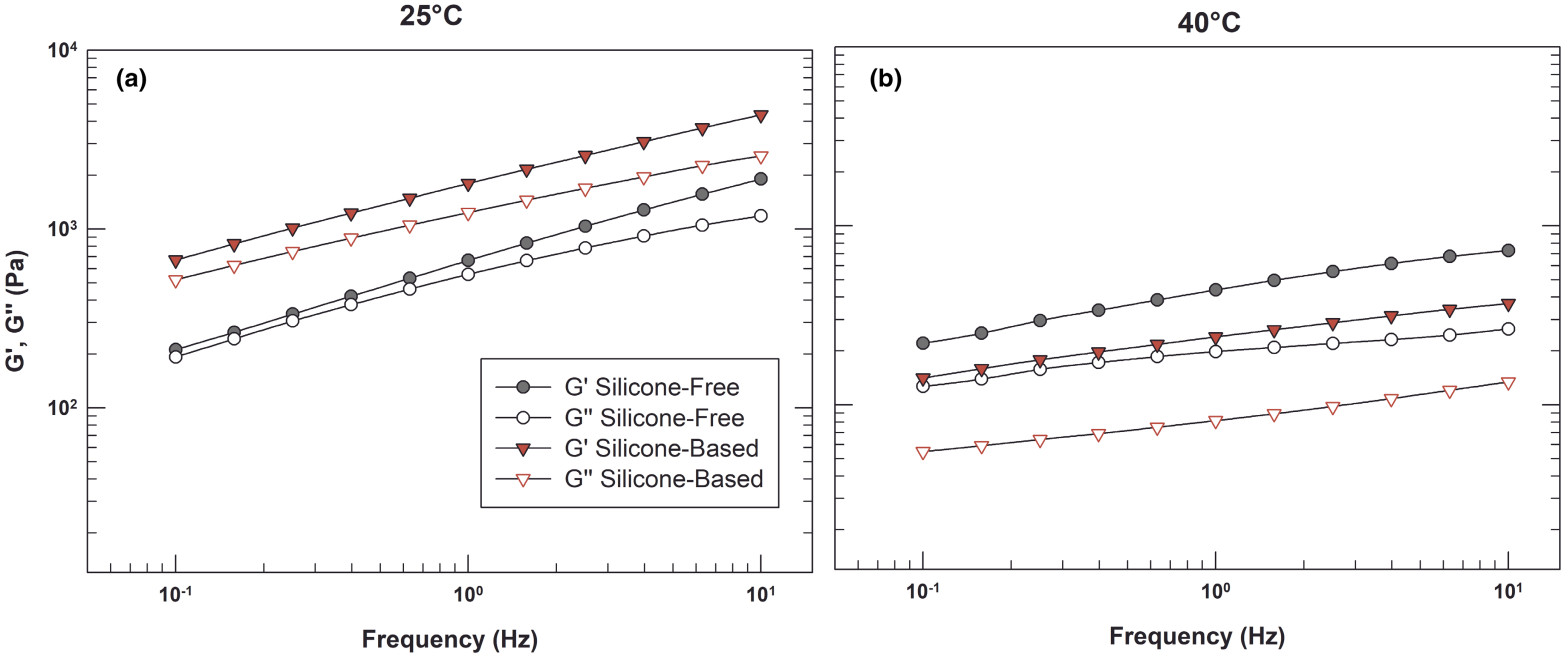
Frequency sweep test under controlled shear stress at 25°C ± 1°C and 40°C ± 1°C on silicone‐based and silicone‐free emulsions. The results are representative of three independent experiments. [Colour figure can be viewed at wileyonlinelibrary.com]

As reported in Figure 6, at room temperature a slight prevalence of G′ above G″ occurred for both samples. The preponderance of elastic modulus values on viscous modulus was clearer in the silicone‐based sample because no superimposing resulted for any value of frequency (triangular symbols) instead of the silicone‐free samples (represented with circular symbols), but it also confirmed the solid‐like nature of both the obtained emulsions.

The analysis carried out at 40°C proved that these mechanical features were maintained in this experimental condition, thus confirming, as already demonstrated, the ability of both emulsions to maintain their internal structure, even in the presence of oscillating stresses. The gap between G′ and G″ values of silicone‐based emulsion is greater than the same parameters of silicone‐free sample, when tested at 40°. This result confirmed that the solid‐like behaviour was maintained and strengthened by warming. Highlighting again that silicones were able to stabilize the resulting products also in the presence of high temperatures.

### In vivo evaluation of emulsions’ effects on skin

**FIGURE 7:**
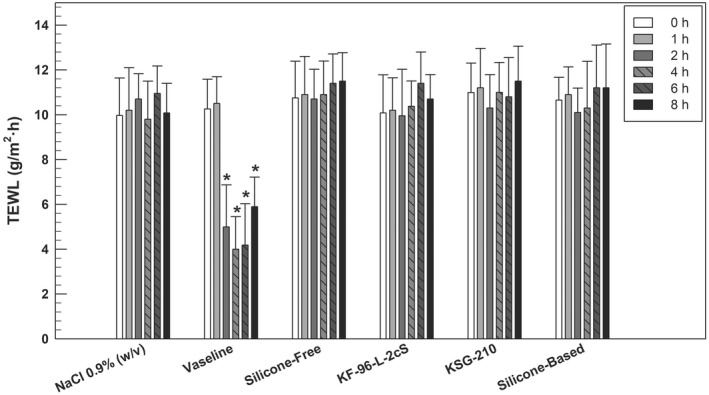
Evaluation of emulsions' skin occlusion properties by recording TEWL values over time, after the application of samples on healthy human volunteers (*n* = 12, totally) in non‐occlusive conditions. Results are expressed as the mean values ± standard deviation. *p < 0.05 compared with the value recorded at time zero (baseline).

Considering that a prolonged occlusion could generate a barrier damage or skin overhydration [53], the potential occlusivity of realized emulsions was assessed by an in vivo study on healthy human volunteers (*n* = 12) by recording the TEWL values after different application times (0, 1, 2, 4, 6 and 8 h) under non‐occlusive experimental conditions. Before starting the study, no significant differences in baseline assessments (time 0) were found in TEWL values for the six tested cutaneous sites. A saline solution (NaCl 0.9% w/v) was used in the study as negative control of occlusion, while Vaseline (or petroleum jelly) was applied on the skin as a positive control of occlusion, as it belongs to an occlusive agent with moisturizing properties [54]. In addition to evaluating the eventual change in TEWL following the application of emulsions prepared with and without the addition of silicones, both volatile dimethicone (KF‐96 L‐2cs) and elastomeric dimethicone (KSG‐210) were tested on the skin as free form. The graph in Figure 7 showed a marked decrease in TEWL values (*p ˂ 0.05) for Vaseline application site starting from the second hour until the end of experiment, confirming the suitability of the in vivo test and the human volunteers' skin reactivity. For all other samples, the TEWL values were absolutely comparable to the saline solution, assessing that neither the two types of silicones in free form, nor the emulsions containing both silicones created conditions of skin occlusion, keeping the trans epidermal water loss into a suitable value range with respect to time zero value of each marked site.

Despite some commercial products were used to induce a specific occlusion of skin [21], often the occlusion is an unwanted condition, and the demonstration of no‐occlusion skin state after the application of these silicone compounds is an important result, because the skin compatibility and tolerability of silicon‐based emulsion were established.

**FIGURE 8:**
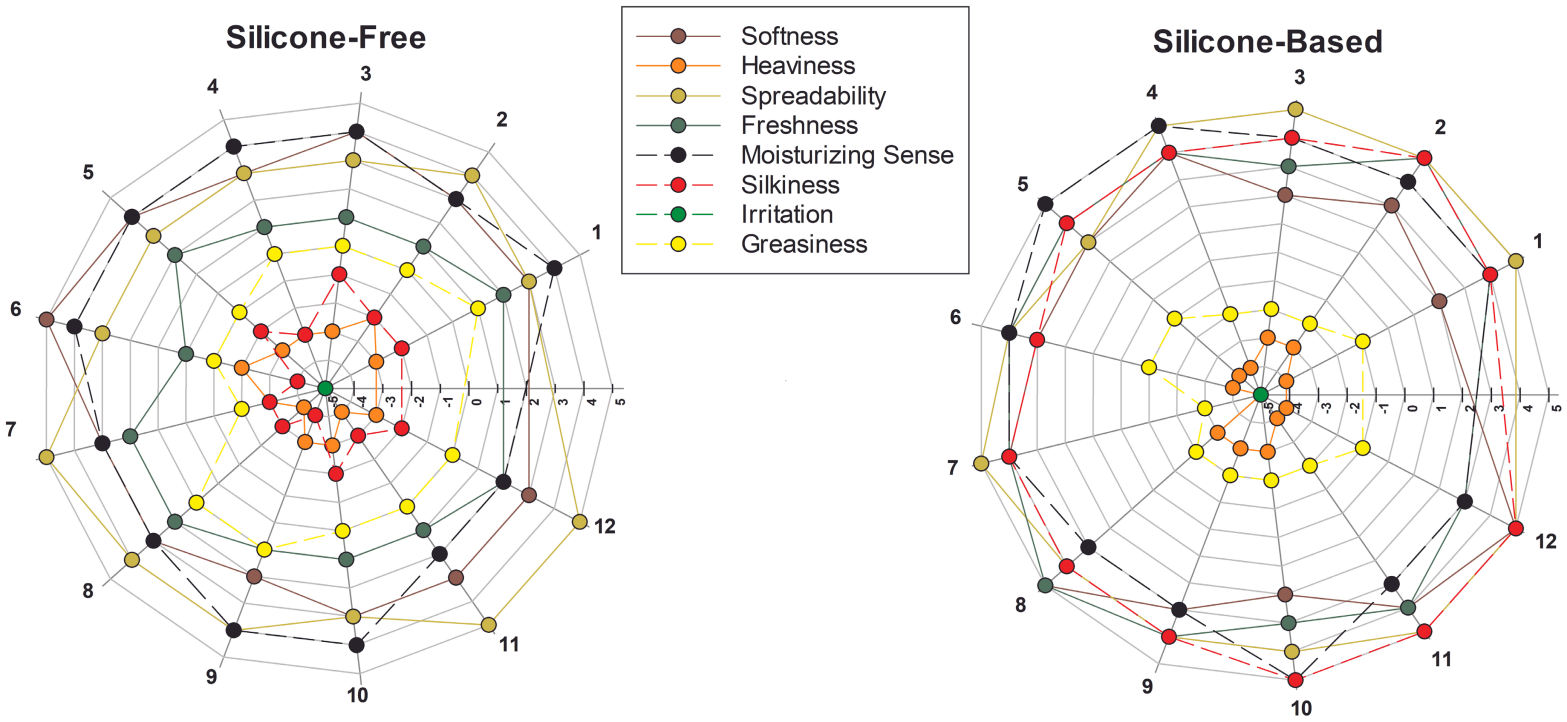
Radar diagrams related to primary (solid lines) and secondary (dashed lines) skin feeling rated by healthy human volunteers (*n* = 12, totally) through an anonymous questionnaire, in single‐blind condition. [Colour figure can be viewed at wileyonlinelibrary.com]

The same volunteers chosen for the in vivo experimentation were asked to fill in an anonymous questionnaire relating to the primary and secondary skin feeling during and following the application of silicone‐free and silicone‐based emulsions on the back of the hand. The procedure was performed under single‐blind conditions, so the candidates were oblivious of the composition of the creams tested. The questionnaires were composed of rating questions on eight specific characteristics of skin feeling, of which four were related to the sensation felt during application and spreading of the topical formulation, while the residual four were referred to posthumous sensations that the formulations left on the skin, after complete absorption. For each feature every candidate released a score value from −5 to +5. Later, the results were collected into radar diagrams (Figure 8), one for each emulsion, to obtain a representative illustration of the candidates' feedback.

Among the obtained ratings, some parameters appeared very different between the two emulsions. For example, the secondary skin feeling property of silkiness was in a negative values range for the silicone‐free emulsion, while all candidates rated it with highest values (+3, +4 and +5) for the silicone‐based emulsion. Also, in the estimation of the greasiness, the emulsion containing the silicones received lower values compared to the silicone‐free emulsion, as found in relation to the heaviness of the cream, but with less marked differences. Especially, the silkiness and the non‐greasy texture are two properties strongly related to the presence of silicones [55]. Other features, such as spreadability or moisturizing sense, were almost overlapped in both diagrams, highlighting no special differences in formulations. Irritation values were considered absent in both cases, as expected for completely non‐toxic elements which composed the emulsions.

## CONCLUSION

Nowadays, silicones are present in several cosmetic formulas, which enter the life of consumers every day as skin protectant or to ameliorate spreading and sensation of skin. They are included in products of daily skin and hair care, as well as in makeup, and in more specific products for treatments of physiological or pathological states of adults and children too. The investigation of the cutaneous effects of these excipients are of great interest, as well as the physico‐chemical and technological features of silicone‐based formulation. The aim of this research work was a full characterization of silicon‐free and silicone‐based emulsions, and the produced results underlined a comparable technological profile of samples containing two different types of silicones, widely findable as cosmetic ingredients, with respect to silicone‐free products. Considering that the feedback from potential consumers is even more important, the different emulsions were tested on human volunteers. They demonstrated their safety through an in vivo study. In particular, TEWL measurements, an essential parameter in defining the integrity and the degree of evaporation of the water from the deep layers of the skin, showed that silicone‐based emulsions were non‐occlusive and they did not alter skin equilibrium, resulting as non‐irritating and breathable cosmetic formulations. Furthermore, the validity of the study was exacerbated by obtaining a direct opinion from potential consumers who, by means of the skin feeling rating, reviewed silicone emulsions more positively than the silicone‐free ones. Silicone‐based emulsions were rated as more pleasant both at the time of application and after complete spreading. These findings corroborated and supported the conscious and thoughtful use of some silicones, which can improve the functionality and even the marketability of cosmetic products.

## CONFLICT OF INTEREST

None.
